# Mitochondrial modulators for obsessive–compulsive and related disorders: a systematic review and meta-analysis

**DOI:** 10.1038/s41398-022-02026-5

**Published:** 2022-06-28

**Authors:** Taro Kishi, Kenji Sakuma, Nakao Iwata

**Affiliations:** Department of Psychiatry, Fujita Health University School of Medicine, Toyoake, Aichi 470-1192 Japan

**Keywords:** Clinical pharmacology, Psychiatric disorders

## Abstract

It remains unclear whether mitochondrial modulators (MMs) are beneficial in the treatment of obsessive–compulsive and related disorders. Thus, in an attempt to answer this clinical question, we performed a systematic review and a random-effects meta-analysis of double-blind, randomized, placebo-controlled trials. The primary outcome was change in overall symptoms as measured using standardized rating scales. Other outcomes were response to treatment; improvement in anxiety-related scales scores, depression-related scale scores, Clinical Global Impression Severity Scale (CGI-S) scores, and Sheehan Disability Scale (SDS) scores; all-cause discontinuation; and individual adverse events. We calculated the standardized mean differences for continuous outcomes and risk ratios for dichotomous outcomes with 95% confidence intervals. We reviewed 17 studies (*n* = 629, 72.62% female; duration = 2–20 weeks; mean age = 30.47 years) of MMs: eicosapentaenoic acid (*K* = 1), folic acid (*K* = 1), lithium (*K* = 1), *N*-acetylcysteine (*K* = 10), inositol (*K* = 3), and silymarin (*K* = 1). MMs outperformed placebo in overall improvement in symptoms (*p* < 0.01) and in improving anxiety-related scale scores (*p* = 0.05). Subgroup analysis of individual MMs revealed that although overall symptoms were better improved by *N*-acetylcysteine (*p* < 0.01) and lithium (*p* = 0.04), no MMs outperformed placebo in terms of improving anxiety-related scale scores. Neither pooled nor individual MMs outperformed placebo in improving response to treatment, depression-related scale scores, CGI-S scores, SDS scores, or all-cause discontinuation. *N*-acetylcysteine was no more associated with a higher incidence of individual adverse events including gastrointestinal symptoms, than placebo. In conclusion, *N*-acetylcysteine was beneficial in the treatment of obsessive–compulsive and related disorders. However, further study with larger samples is necessary to confirm this finding.

## Introduction

Obsessive–compulsive disorder (OCD), characterized by the presence of obsessions or compulsions, or both [1], is a common chronic mental disorder; the lifetime prevalence of OCD among adults in the United States was 2.3% [2]. Both pharmacotherapy, such as antidepressants, and psychotherapeutic methods, such as exposure and response prevention therapy, are among the primary treatments for OCD [3]. However, not all individuals with OCD respond to these treatments; thus, new therapeutic agents with new pharmacological mechanisms of action that differ from those of conventional psychotropic drugs are needed.

Oxidative stress and free radicals, inflammation, and mitochondrial dysfunction have recently been shown to play key roles in the development of OCD [4, 5]. Mitochondrial dysfunction and oxidative stress are tightly dependent on each other [4, 5]. For example, a positron emission tomographic study demonstrated inflammation in not only the basal ganglia but also in the cortico-striato-thalamo-cortical circuit in individuals with OCD [6]. A cross-sectional study showed that serum levels of 8-hydroxydeoxyguanosine, which is a marker of oxidative DNA damage, were significantly higher in individuals with OCD than in those without OCD and were lower in the patients who received treatment for OCD [7].

To date, 17 double-blind, randomized, placebo-controlled trials of mitochondrial modulators such as *N*-acetylcysteine and inositol in treating OCD have been conducted (Table 1). Of these, ten focused on *N*-acetylcysteine, a precursor of endogenous antioxidant glutathione that has antioxidant, anti-inflammatory, and neuroprotective properties [8, 9]. *N*-acetylcysteine may also have mitochondrial biogenesis effects [10]. Results regarding the efficacy of mitochondrial modulators against OCD were noted to be inconsistent among the double-blind, randomized, placebo-controlled trials (Table 1), and so it remains unclear whether mitochondrial modulators are beneficial in the treatment of OCD. The discrepancy in results may be attributable to the smallness of the samples in these trials. A meta-analysis produces a weighted summary result, in which more weight is given to larger studies. Combining results from more than one study has the advantage of increasing statistical power, which is often inadequate in studies with small sample sizes [11]. Therefore, we conducted a systematic review and meta-analysis of these studies to examine the efficacy, tolerability, and safety of mitochondrial modulators in the treatment of mood disorders in individuals with OCD and OCD-related disorders.

**Table 1:** Characteristics of patients and treatments in double-blinded, randomized, placebo-controlled trials included in this study.

	Disease	Country	Fund	Total *n*	Duration	Age (mean ± SD, years)	Female (%)	Diagnosis	Inclusion	Intervention	Control	Efficacy for OCD symptoms^a^
Afshar 2012 NAC	Adults OCD	Iran	Academia	48	12	30.93 ± 4.99	75.00	DSM-IV	PT had failed to clinically respond to at least 12 weeks of high-dose treatment with a SSRI or clomipramine (Y-BOCS: 16 or greater)	NAC 2400 mg/d + SRI	PLA + SRI	NAC > PLA
Bloch 2013 NAC	Children TRI	USA	Academia	39	12	13.56 ± 2.76	87.18	DSM-IV		NAC 2400 mg/d + TAU	PLA + TAU	NAC = PLA
Costa 2017 NAC	Adults OCD	Brazil	Academia	40	16	38 ± 10.8	47.50	DSM-IV	PT had failed to clinically respond to at least 12 weeks of maximum recommended or tolerated dose of SSRI or clomipramine (Y-BOCS: 16 or greater OR only compulsions: 10 or greater and CGI-S 4 or greater)	NAC 3000 mg/d + TAU	PLA + TAU	NAC = PLA
Fux 1996 INO	Adults OCD	Israel	Academia	15	6	33.7	61.54	DSM-III-R	PT had failed to clinically respond to SSRI or clomipramine. Patients were drug free for at least one week before beginning the trial.	INO 18 g/d	PLA	INO > PLA
Fux 1999 INO	Adults OCD	Israel	Academia	13	6	30.3 ± 9	80.00	DSM-IV	PT had failed to clinically respond to SSRI or clomipramine.	INO 18 g/d + SRI	PLA + SRI	INO = PLA
Fux 2004 EPA	Adults OCD	Israel	Academia	11	6	33.5 ± 5	72.73	DSM-IV	PT had failed to adequately respond to at least 8 weeks of treatment with a stable maximally tolerated dose of SSRI.	EPA 2 g/d + SSRI	PLA + SSRI	EPA = PLA
Ghanizadeh 2017 NAC	Children OCD	Iran	Academia	34	10	16.27 ± 3.18	48.28	DSM-IV-TR	PT failed to respond to at least a previous a trial of an SSRI. The patients did not receive any serotonin reuptake inhibitors with adequate therapeutic duration and dosage in the last one month before randomization.	NAC 1200–2400 mg/d + citalopram 20–40 mg/day	PLA + citalopram 20 to 40 mg/day	NAC > PLA
Grant 2009 NAC	Adults TRI	USA	Academia	50	12	34.3 ± 12.1	90.00	DSM-IV-TR		NAC 2400 mg/d + TAU	PLA + TAU	NAC > PLA
Grant 2016 NAC	Adults EXC	USA	Academia	66	12	34.8 ± 11	87.88	DSM-5		NAC 1200–3000 mg/d + TAU	PLA + TAU	NAC > PLA
Grant 2019 SIL	Children and Adults TRI	USA	Academia	20	6	27.9 ± 11.5	95.00	DSM-5		milk thistle 300–600 mg/d + TAU	PLA + TAU	SIL = PLA
Leppink 2017 INO	Adults TRI	USA	Academia	38	10	28.9 ± 11.4	92.11	DSM-5		INO 6–18 g/d + TAU	PLA + TAU	INO = PLA
Li 2020 NAC	Children OCD	USA	Academia	11	12	11.93 ± 2.9	72.73	DSM-5	CY-BOCS: 16 or greater	NAC 2700 mg/d + TAU	PLA + TAU	NAC > PLA
McDougle 1991 LIT	Adults OCD	USA	Academia	20	2	34.48 ± 11.73	80.00	DSM-III-R	PT had failed to adequately respond to at least 8 weeks of treatment with fluvoxamine (Y-BOCS: 16 or greater. CGI-I: 3 or greater).	LIT 0.5–1.2 mmol/L + fluvoxamine (286.4 ± 23.4 mg/d)	PLA + fluvoxamine (277.8 ± 44.1mg/d)	LIT > PLA
Paydary 2016 NAC	Adults OCD	Iran	Academia	46	10	33.38 ± 11.22	75.00%	DSM-IV-TR	Y-BOCS: 21 or greater	NAC 2000 mg/ d + fluvoxamine 200 mg/d	PLA+fluvoxamine 200 mg/d	NAC > PLA
Sarris 2015 NAC	Adults OCD	Australia	Academia	44	16	37 ± 12.17	45.45	DSM-5	Y-BOCS: 16 or greater. PT be on either no treatment or a stable treatment regimen for a minimum of 4 weeks of current treatment and a minimum of 12 weeks if this is their first OCD treatment	NAC 3000 mg/d + TAU	PLA + TAU	NAC = PLA
Sarris 2022 NAC	Adults OCD	Australia	Academia	98	20	32 (median)	67.42	DSM-5	Y-BOCS: 16 or greater. PT be on a stable treatment regimen for a minimum of 8 weeks of current treatment and a minimum of 12 weeks if this is their first OCD treatment	NAC 2000–4000 mg/d + TAU	PLA + TAU	NAC = PLA
Tural 2019 FA	Adults OCD	Turkey	Industry	36	12	32.6 ± 10.9	61.11	DSM-IV-TR		FA 5 mg/d + fluoxetine 40 mg/d	PLA + fluoxetine 40 mg/d	FA = PLA

*CGI-I* Clinical Global Impression–Improvement Scale, *CGI-S* Clinical Global Impression Severity Scale, *CY-BOCS* Children’s Yale–Brown Obsession Compulsion Scale, *DSM* Diagnostic and Statistical Manual of Mental Disorders, *EPA* eicosapentaenoic acid, *EXC* excoriation disorder, *FA* folic acid, *INO* inositol, *LIT* lithium, *n* number of patients, *NAC* N-acetylcysteine, *OCD* obsessive–compulsive disorder, *PLA* placebo, *PT* patient, *SD* standard deviation, *SIL* silymarin, *SRI* serotonin reuptake inhibitor, *SSRI* selective serotonin reuptake inhibitor, *TAU* treatment as usual, *TRI* trichotillomania, *Y-BOCS* Yale–Brown Obsession Compulsion Scale.

^a^A > B means that A was superior to B; A = B means that A was similar to B.

## Subjects and methods

The systematic review and meta-analysis were performed according to the Preferred Reporting Items for Systematic Reviews and Meta-Analysis (PRISMA) guidelines (Appendix S1) [12] and were registered with the Open Science Framework (https://osf.io/h4ypt). The literature search, data transfer accuracy, and statistics were each double-checked by at least two of the authors.

### Search strategy, inclusion criteria, and data extraction

A systematic literature review was performed to characterize patients, interventions, comparisons, and outcome strategies. Patients were adults and children with OCD and OCD-related disorders (body dysmorphic disorder, hoarding disorder, trichotillomania, or excoriation [skin picking] disorder) [1]. Interventions consisted of mitochondrial modulator therapy: *N*-acetylcysteine, acetyl-l-carnitine, *S*-adenosylmethionine, coenzyme Q10, alpha-lipoic acid, creatine monohydrate, vitamin C, vitamin D, vitamin E, melatonin, omega-3 polyunsaturated fatty acids, inositol, silymarin, folic acid, resveratrol, sulforaphane, and lithium [4, 13–15]. The control conditions involved placebo. Outcomes are listed in the following section.

We identified eligible studies indexed in Embase, PubMed, and the Cochrane Library databases, published in any language, and containing data from the inception of the study to March 14, 2022 (Fig. S1). The search terms in PubMed and the Cochrane Library databases included “placebo”; “random*”; a specific disorder (one of the following: obsessive–compulsive disorder, OCD, body dysmorphic disorder, hoarding disorder, trichotillomania, excoriation, or body-focused repetitive behaviors); and one specific mitochondrial modulator (*N*-acetylcysteine, acetyl-l-carnitine, *S*-adenosylmethionine, coenzyme Q10, alpha-lipoic acid, creatine monohydrate, vitamin C, vitamin D, vitamin E, melatonin, omega-3 polyunsaturated fatty acids, docosahexaenoic acid, eicosapentaenoic acid, inositol, silymarin, folic acid, resveratrol, sulforaphane, or lithium). The search terms for Embase included names of the aforementioned disorders, randomized controlled trial and names of the aforementioned individual mitochondrial modulators. The authors also searched the ClinicalTrials.gov (http://clinicaltrials.gov/) and the International Clinical Trials Registry Platform (http://www.who.int/ictrp/en/) to ensure that the search was comprehensive and to minimize publication bias. The reference lists of the retrieved publications were also searched for additional relevant published and unpublished studies, including conference abstracts.

### Outcomes and data synthesis

We extracted data from the included articles and entered the data in spreadsheets. We first reviewed the study design and the characteristics of patients and treatments in the studies.

The primary outcome was change in overall symptoms, measured using standardized rating scales such as the Yale–Brown Obsession Compulsion Scale [16], the Massachusetts General Hospital Hair Pulling Scale [17], and the Yale–Brown Obsessive–Compulsive Scale Modified for Neurotic Excoriation [18]. Other outcomes included study-defined response to treatment; scores on anxiety-related scales, such as the Hamilton Anxiety Rating Scale [19] and Beck Anxiety Inventory [20]; scores on depression-related scales, such as the Hamilton Depression Rating Scale [21], the Children’s Depression Inventory [22], the Beck Depression Inventory [23], and the Montgomery–Åsberg Depression Rating Scale [24]; scores on the Clinical Global Impression Severity Scale (CGI-S) [25]; scores on the Sheehan Disability Scale (SDS) [26]; rate of all-cause discontinuation of treatment; and incidence of individual adverse events.

### Statistical analysis

**Fig. 1:**
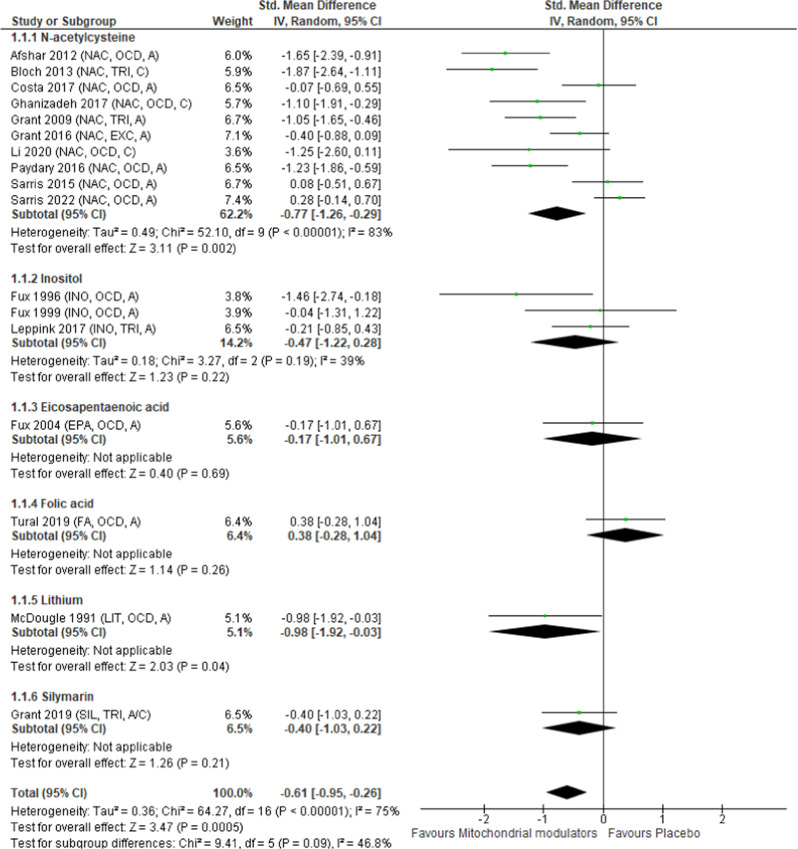
Forest plot for overall symptoms. Studies are listed by names of the first authors and year of publication, with abbreviations for drugs listed in parentheses (**A**, **B**, **C**). **A** Listing by mitochondrial modulator name. EPA eicosapentaenoic acid, FA folic acid, INO inositol, LIT lithium, NAC N-acetylcysteine, SIL silymarin. **B** Listing by diagnosis. EXC excoriation disorder, OCD obsessive–compulsive disorder, TRI trichotillomania. **C** Listing according to adults (**A**) or children (**C**).

To perform the pairwise meta-analysis, we used Review Manager software (version 5.4.1; Copenhagen, The Nordic Cochrane Centre) [27]. Because of the potential for heterogeneity across the included studies, we used a random-effects model [28]. We calculated the standardized mean differences (SMDs) for continuous outcomes and risk ratios for dichotomous outcomes with 95% confidence intervals (CIs). Heterogeneity was tested with the I^2^ statistic, whereby I^2^ of ≥50% indicated considerable heterogeneity [11]. As shown in Fig. 1, we found considerable heterogeneity for the primary outcome in our meta-analysis. Only *N*-acetylcysteine and lithium outperformed placebo in improving overall symptoms. Because only one study focused on lithium, we conducted a sensitivity analysis to include only studies of *N*-acetylcysteine, using meta-regression to evaluate the following potential confounding factors: (1) mean age (years), (2) diagnosis (OCD or OCD-related disorders), (3) study duration (in weeks), (4) total number of individuals, and (5) percentage of female participants. Because reports of studies of all mitochondrial modulators other than *N*-acetylcysteine did not include sufficient data for us to perform a meta-analysis regarding safety outcomes, we conducted these meta-analyses for only studies of *N*-acetylcysteine. We used a funnel plot and Egger’s regression to assess whether small-study effects were attributable to publication bias [29]. For the meta-regression analysis, constructing the funnel plot, and assessing results of Egger’s regression, we used the Comprehensive Meta-Analysis software version V3 (Biostat Inc., Englewood, NJ, USA). We assessed the methodological quality of the included articles according to the Cochrane risk of bias criteria [11].

## Results

### Results of the literature search

Of the 119 retrieved publications, 37 duplicates and 64 publications based on an abstract or a title review were excluded. In addition, one post hoc study [30] was excluded after a full-text review (Fig. S1). No additional studies were retrieved from the clinical trial registries or in manual searches with reference to the previous systematic reviews [31–38]. Ultimately, we reviewed 17 studies involving a total of 629 patients [39–55].

### Study characteristics

Table 1 lists the characteristics of each study and the characteristics of patients included in each study. Eicosapentaenoic acid (1 study) [41], folic acid (1 study) [43], lithium (1 study) [55], *N*-acetylcysteine (10 studies) [44–47, 49–54], inositol (3 studies) [39, 40, 48], and silymarin (1 study) [42] were the mitochondrial modulators studied. Twelve studies included individuals with OCD [39–41, 43–47, 50, 51, 53, 55], four others included individuals with trichotillomania [42, 48, 52, 54], and the remaining study included individuals with excoriation disorder [49]. Thirteen studies included adults [39–41, 43, 44, 47–51, 53–55], three others included children [45, 46, 52], and one included both adults and children [42]. In all studies except one of inositol [39], the investigators assessed mitochondrial modulator add-on to conventional treatment, such as pharmacotherapy (in most studies, serotonin reuptake inhibitors) or psychotherapy. Patients’ mean age was 30.47 years, 72.62% of participants were female, and study duration ranged from 2 to 20 weeks. In two studies of inositol [39, 40], that is, one of eicosapentaenoic acid [41] and one of silymarin [42], a crossover design was used. For the two inositol studies, data from the first treatment phase could be used to analyze efficacy outcomes, although the data were from a complete analysis. Except for one study of folic acid [43], none of the trials was sponsored by pharmaceutical companies. The methodological quality of most studies was high (Fig. S2).

### Overall symptoms

In all 17 studies, the mitochondrial modulators outperformed placebo (*n* = 632, SMD = −0.61, 95% CI = −0.95 to −0.26, *p* < 0.01, *I*^2^ = 75%; Table 2, Fig. 1). According to subgroup analysis of individual mitochondrial modulators, placebo was outperformed by *N*-acetylcysteine (in 10 studies involving 453 patients; SMD = −0.77, 95% CI = −1.26 to −0.29, *p* < 0.01, *I*^2^ = 83%) and by lithium (in 1 study involving 20 patients; SMD = −0.98, 95% CI = −1.92 to −0.03, *p* = 0.04, *I*^2^ was not applicable; Fig. 1). When study results were pooled together, the *p* value of Egger’s regression for the outcome was 0.15, and the funnel plot was visually symmetric (Fig. S3).

**Table 2:** Efficacy, acceptability, and tolerability outcomes: pooled mitochondrial modulators versus placebo.

Continuous variables					
	*N*	*n*	SMD (95% CI)	*P*-value	*I*^2^
Overall symptoms	17	632*	−0.61 (−0.95, −0.26)	<0.01	75%
Anxiety symptoms	9	411	−0.20 (−0.39, −0.00)	0.05	0%
Depression symptoms	10	450	0.00 (−0.32, 0.32)	0.99	63%
CGI-S scores	7	364	−0.20 (−0.64, 0.23)	0.36	76%
SDS scores	4	245	−0.25 (−0.50, 0.00)	0.05	0%

**Dichotomous variables**					
	***N***	***n***	**RR (95% CI)**	***p*** **value**	***I***^2^
Response to treatment	13	457	1.38 (0.98, 1.95)	0.07	7%
All-cause discontinuation	11	490	0.76 (0.43, 1.33)	0.33	27%

* In two studies of inositol [39, 40], that is, one of eicosapentaenoic acid [41] and one of silymarin [42], a crossover design was used. For the two inositol studies, data from the first treatment phase could be used to analyze efficacy outcomes, although the data were from a complete analysis.

*95%* *CI* 95% confidence interval, *CGI-S* Clinical Global Impression Severity Scale, *N* number of studies, *n* number of patients, *RR* risk ratio, *SDS* Sheehan Disability Scale, *SMD* standardized mean difference.

A meta-regression analysis demonstrated no associations between effect size and any potential confounding factors (Table 3). Although the funnel plot for only *N*-acetylcysteine studies was visually symmetric (Fig. S4), the p value of Egger’s regression for the meta-analysis that included only studies of *N*-acetylcysteine were 0.03.

**Table 3:** Meta-regression analysis for overall symptoms: N-acetylcysteine versus placebo.

Covariate	Coefficient	Standard	95% CI	*Z* value	*P* value
Mean age (year)	0.04	0.04	−0.03, 0.11	1.18	0.24
Diagnosis (OCD or OCD-related disorders)	−0.90	0.73	−2.34, 0.54	−1.23	0.22
Study duration (in weeks)	0.02	0.13	−0.24, 0.27	0.13	0.90
Total number of individuals	0.00	0.03	−0.05, 0.06	0.09	0.93
Percentage of female participants	−0.04	0.02	−0.07, 0.00	−1.89	0.06

*95% CI* 95% confidence interval, *OCD* obsessive–compulsive disorder.

### Other efficacy outcomes and all-cause discontinuation of therapy

**Fig. 2:**
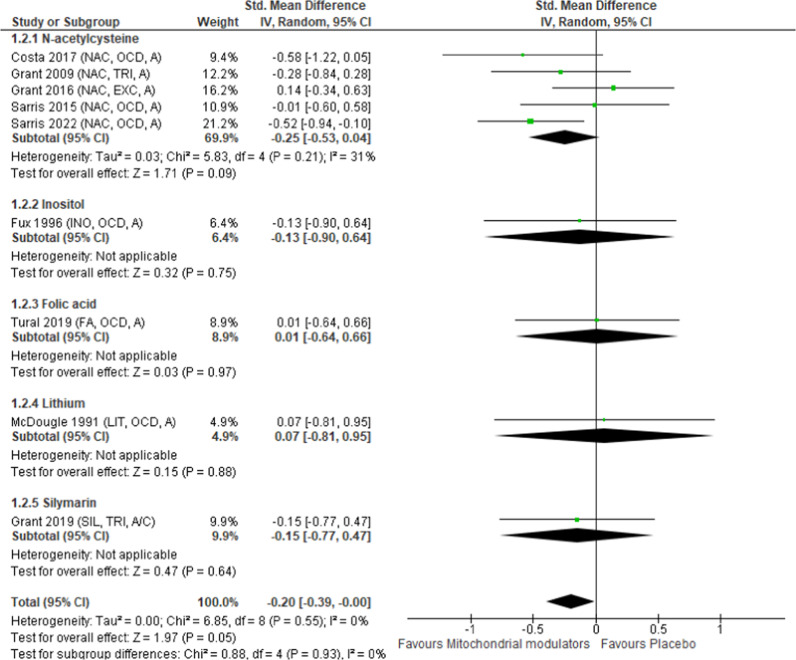
Forest plot for anxiety symptoms. Studies are listed by names of the first authors and year of publication, with abbreviations for drugs listed in parentheses (**A**, **B**, **C**). **A** Listing by mitochondrial modulator name. FA folic acid, INO inositol, LIT lithium, NAC N-acetylcysteine, SIL silymarin. **B** Listing by diagnosis. EXC excoriation disorder, OCD obsessive–compulsive disorder, TRI trichotillomania. **C** Listing according to adults (**A**) or children (**C**).

When study results were pooled together, mitochondrial modulators outperformed placebo in improving anxiety symptoms in 9 studies involving 411 patients (SMD = −0.20, 95% CI = −0.39, −0.00, *p* = 0.05, *I*^2^ = 0%; Table 2, Fig. 2). Subgroup analysis of individual mitochondrial modulators revealed that *N*-acetylcysteine was marginally superior to placebo in improving anxiety symptoms (*p* = 0.09), but other individual mitochondrial modulators did not outperform placebo (Fig. 2). Pooled mitochondrial modulators have marginally outperformed placebo in improving, SDS scores (*p* = 0.05; Table 2, Fig. S5) and response to treatment (*p* = 0.07; Table 2, Fig. S6). Subgroup analysis of individual mitochondrial modulators revealed that *N*-acetylcysteine marginally outperformed placebo in improving SDS scores (*p* = 0.05; Fig. S5) and response to treatment (*p* = 0.09; Fig. S6). Neither pooled nor individual mitochondrial modulators outperformed placebo in terms of improving CGI-S scores and depression-related scale scores or rates of all-cause discontinuation of treatment (Table 2, Figs. S7, S8 and S9).

### Safety outcomes

In comparison with placebo, *N*-acetylcysteine was not associated with a higher incidence of insomnia, somnolence, fatigue, dizziness, headache, dry mouth, nausea/vomiting, diarrhea, constipation, or rash (Table 4).

**Table 4:** Safety outcomes: N-acetylcysteine versus placebo.

	*N*	*n*	RR (95% CI)	*P* value	*I*^2^
Insomnia	2	73	1.35 (0.32, 5.82)	0.68	7%
Somnolence	3	120	1.42 (0.72, 2.79)	0.31	0%
Fatigue	3	113	1.22 (0.43, 3.48)	0.70	35%
Dizziness	4	186	1.38 (0.70, 2.74)	0.35	0%
Headache	3	120	0.92 (0.44, 1.91)	0.81	0%
Dry mouth	3	146	0.95 (0.39, 2.34)	0.91	0%
Nausea/vomiting	7	323	1.15 (0.53, 2.50)	0.72	46%
Diarrhea	6	228	1.96 (0.78, 4.92)	0.15	0%
Constipation	4	186	0.88 (0.42, 1.84)	0.74	0%
Rash	3	84	2.90 (0.49, 17.29)	0.24	0%

*95% CI* 95% confidence interval, *N* number of studies, *n* number of patients, *RR* risk ratio.

## Discussion

This is the first systematic review and meta-analysis in which the efficacy, acceptability, and safety of mitochondrial modulators (eicosapentaenoic acid, folic acid, inositol, lithium, *N*-acetylcysteine, and silymarin) were compared with those of conventional treatment for OCD and OCD-related disorders. Except for one study of inositol [39], all studies involved mitochondrial modulator add-on to conventional treatment, such as serotonin reuptake inhibitors. As a group, mitochondrial modulators have significantly improved overall symptoms of OCD and anxiety. In particular, lithium and *N*-acetylcysteine significantly improved overall OCD symptoms, and *N*-acetylcysteine marginally improved anxiety symptoms. As a group, mitochondrial modulators also marginally outperformed placebo in improving SDS scores and response to treatment. In particular, *N*-acetylcysteine marginally improved SDS scores and response to treatment. Moreover, *N*-acetylcysteine did not carry any risk of adverse events. Thus, *N*-acetylcysteine as an antidepressant adjunct might be a novel treatment option for individuals with OCD and OCD-related disorders who received conventional treatment.

Our meta-analysis showed that the overall effect size of *N*-acetylcysteine was moderate (SMD = −0.77) and comparable with the antidepressant effect sizes of aripiprazole (SMD = −1.35), haloperidol (SMD = −0.82), and risperidone (SMD = −0.59), as indicated by a previous meta-analysis [56]. Lithium was able to improve overall OCD symptoms, but because only one study involving lithium (in 20 patients) was included in our systematic review, further study with larger samples is needed to confirm our findings.

We found considerable heterogeneity in the results of *N*-acetylcysteine with regard to the primary outcome, but our meta-regression analysis demonstrated no associations between effect size and any confounding factors. The funnel plot was visually symmetric; however, because the *p* value of Egger’s regression was 0.03, small-study effects might have influenced this result [29]. Another limitation of our study was that the trials included in our meta-analysis were of short duration. Other limitation of our study was that there was only one trial for eicosapentaenoic acid, folic acid, lithium, and silymarin. Therefore, longer studies, as well as larger samples, are necessary to confirm our results.

In conclusion, *N*-acetylcysteine was beneficial in the treatment of OCD and OCD-related disorders. However, because all studies included in our systematic review had small sample sizes, small-study effects might have influenced our findings. To obtain robust evidence, further studies with larger samples are needed.

## Supplementary information

Supplementary materials

## Data Availability

Data used for the current study were reported in articles as cited in this paper.
